# Adipose-derived mesenchymal stem cells may reduce intestinal epithelial damage in ulcerative colitis by communicating with macrophages and blocking inflammatory pathways: an analysis *in silico*

**DOI:** 10.18632/aging.203964

**Published:** 2022-03-22

**Authors:** Nan Zhang, Yixuan Chen, Chengyu Huang, Mengxin Wei, Ting Li, Yufeng Lv, Qiong Song, Shaowen Mo

**Affiliations:** 1 YuanDong International Academy of Life Sciences, Nanning 530000, Guangxi, China; 2 Department of Oncology, Foresea Life Insurance Guangxi Hospital, Nanning 530000, Guangxi, China; 3 Chinese Academy of Science Center for Excellence in Brain Science and Intelligence Technology, Chinese Academy of Science, Shanghai 200031, China

**Keywords:** ulcerative colitis, inflammatory disease, colonic mucosa, submucosa, pathogenesis

## Abstract

Ulcerative colitis is a chronic, non-specific inflammatory disease that affects mainly the colonic mucosa and submucosa. The pathogenesis of ulcerative colitis is unclear, which limits the development of effective treatments. In this study, single-cell sequencing data from 18 ulcerative colitis samples and 12 healthy controls were downloaded from the Single Cell Portal database, cell types were defined through cluster analysis, and genes in each cluster that were differentially expressed in ulcerative colitis were identified. These genes were enriched in functional pathways related to apoptosis, immunity and inflammation. Analysis using iTALK software suggested extensive communication among immune cells. Single-cell sequencing data from adipose-derived mesenchymal stem cells from three healthy female donors were obtained from the Sequence Read Archive database. The SingleR package was used to identify different cell types, for each of which a stemness score was calculated. Pseudotime analysis was performed to infer the trajectory of cells. SCENIC software was used to identify the gene regulatory network in adipose-derived mesenchymal stem cells, and iTALK software was performed to explore the relationship among macrophages, adipose-derived mesenchymal stem cells and enterocytes. Molecular docking confirmed the possibility of cell-cell interactions via binding between surface receptors and their ligands. The bulk data were downloaded and analyzed to validate the expression of genes. Our bioinformatics analyses suggest that ulcerative colitis involves communication between macrophages and enterocytes via ligand-receptor pairs. Our results further suggest that adipose-derived mesenchymal stem cells may alleviate ulcerative colitis by communicating with macrophages to block inflammation.

## INTRODUCTION

Ulcerative colitis (UC) is a chronic, non-specific inflammatory disease that affects mainly the colonic mucosa and submucosa [1]. UC tends to recur and often progresses to cancer [2]. Current treatments include aminosalicylic acid, immunosuppressants and adrenal glucocorticoids, but they are often ineffective and the disease can recur [3].

The pathogenesis of UC is unclear. Pathological examination of intestinal tissues during active UC shows extensive damage to intestinal epithelial cells as well as diffuse inflammation [2, 4]. The intestinal mucosal acts as an immune and mechanical barrier [5] that maintains the stability of the intestinal flora and host immune tolerance toward intestinal microbes [6]. The chronic inflammation in UC can weaken the tight junctions between epithelial cells [7], leading to the destruction of the mucus layer on the surface of the intestinal epithelium [8]. Apoptosis and autophagy may also contribute to damage of the intestinal mucosa in UC [9].

Immune cells appear capable of influencing the course of UC. The subtype of monocytes called macrophages regulate immune responses in the intestinal microenvironment in UC [10]. Macrophages remove apoptotic cells [11] and regulate inflammatory processes [9]. Adipose-derived mesenchymal stem cells (ADMSCs) regulate macrophage function, and they down-regulate pro-inflammatory factors (INFγ, IL-6 and IL-8) while up-regulating anti-inflammatory factors (IL-10, IL-4), thereby weakening the local inflammatory response [12]. Clarifying the roles and interactions of macrophages and ADMSCs in UC may help clarify how the disease occurs and progresses, which may lead to therapeutic targets.

In this bioinformatics study, we found evidence that macrophages may damage the intestinal mucosal barrier by promoting inflammation and intestinal epithelial cell apoptosis/autophagy, likely contributing to UC. We found evidence that, conversely, ADMSCs may communicate with macrophages to block inflammation and thereby alleviate UC.

## MATERIALS AND METHODS

### Single cell data collection and quality control

Single-cell RNA sequencing data from colon biopsies of 18 patients with UC and 12 healthy individuals were collected from the Single Cell Portal database (accession number SCP259) [1]. Single-cell RNA sequencing data from ADMSCs from thigh source of three healthy female donors were obtained from the Sequence Read Archive database (accession number SRP148833) [13].

Sequencing data were subjected to quality control based on the following criteria [14]: gene number between 200 and 6000, unique molecular identifiers (UMI) count > 1000, and mitochondrial gene percentage < 0.1. All 23 samples with sequencing data were used for cell-clustering analysis.

### Dimensional reduction, clustering and cell type identification

The most variable genes in single cells were identified as described [15]. In brief, the average expression and dispersion of each gene were calculated, then the genes were assigned to eight bins based on their expression. The “NormalizeData” function in Seurat [14] was used to normalize the expression matrix of single cells. The expression matrix was multiplied by 10000 using the “LogNormalize” function, then divided by the size of the total library, so that different cells could be compared. The expression levels of highly variable genes were scaled and centered using the “ScaleData” function in order to exclude the influence of mitochondrial genes and the total number of molecules detected within a cell.

Data were visualized in two dimensions using the “uniform manifold approximation and projection for dimension reduction” (UMAP) method. The SingleR package in R (version 0.2.2) was used to independently infer the cell source and identify the type of each single cell, based on the “Immgen” data set [15]. The “Findallmarkers” function in Seurat version 3.1.2 was used to identify differentially expressed genes (DEGs) in UC. DEGs were defined as those showing |log_2_(fold change)| > 1 and P < 0.05 with respect to controls.

To uncover the potential biological significance of DEGs, their enrichment in functional pathways was examined using the Kyoto Encyclopedia of Genes and Genomes (KEGG) within the “clusterprofiler” package in R [16]. P < 0.05 was considered to indicate enrichment.

### Stemness score

The “stemness” gene set was downloaded from the Molecular Signatures database (https://www.gsea-msigdb.org/gsea/msigdb) [17]. Gene set variation analysis was performed using the “GSVA” package in R in order to estimate variation in stemness across different cell types in an unsupervised manner [18].

### Pseudotime analysis

Monocle 3 was used to simulate an evolutionary trajectory through pseudotime [15]. The “importCDS” function in Monocle was used to convert the original count in the Seurat object into the “CellDataSet” data set, and the “differentialGeneTest” function was used to identify genes that may help identify genes whose expression changes across pseudotime (q_val_ < 0.01). The “dimension reduction” function was used for clustering, while the “orderCells” function was used to infer the trajectory based on default parameters. Gene expression was mapped using the “plot_genes_pseudotime” function.

### Cell-cell crosstalk between cell clusters

The iTALK package in R [19] was used to investigate cell-cell crosstalk between cell clusters. Briefly, the top 50% of highly expressed genes in each cell cluster were matched to the 2,648 non-redundant ligand-receptor pairs included in the iTALK package. These pairs fall into four categories, based on whether the ligand serves as a checkpoint protein, cytokine, growth factor, or “other” protein. The top 20 ligand-receptor pairs for each type were visualized as a ligand-receptor interaction network.

### Gene regulatory network and regulons

We used a modified version of the “Single-Cell Regulatory Network Inference” (SCENIC) approach [20, 21] to construct a gene regulatory network from the single-cell RNA sequencing data [22]. First, co-expression modules of transcription factors (TFs) and their potential target genes were identified. Second, the most likely target genes were identified based on enrichment of the appropriate binding motifs in the TFs. The resulting regulons of TFs with their most likely target genes were assigned a “regulon activity score” (RAS) in each single cell, based on the area under the receiver operating characteristic curve.

### The bioinformatical analysis on the bulk data level

The microarray data of UC patients and controls have been downloaded from the Gene Expression Omnibus (GEO) database (accession number: GSE38713, the platform: GPL570) [23]. The bulk data from intestinal mucosa of 13 healthy controls and 30 UC patients. The differentially expressed genes were identified using the Linear Models for Microarray data (limma) package [24] in R software. P < 0.05 was considered to indicate a statistically significant difference.

### Molecular docking

Molecular docking studies of different cell types via surface receptors and ligands were performed using Hex8.0.0.0 software [25] and protein crystal structures available in the Protein Database (https://www.rcsb.org/pages/contactus) [26]. Binding energy < 0 was taken to indicate possible binding. Potential complexes were visualized with Pymol software [27].

## RESULTS

### Total cellular landscape in UC

**Figure 1:**
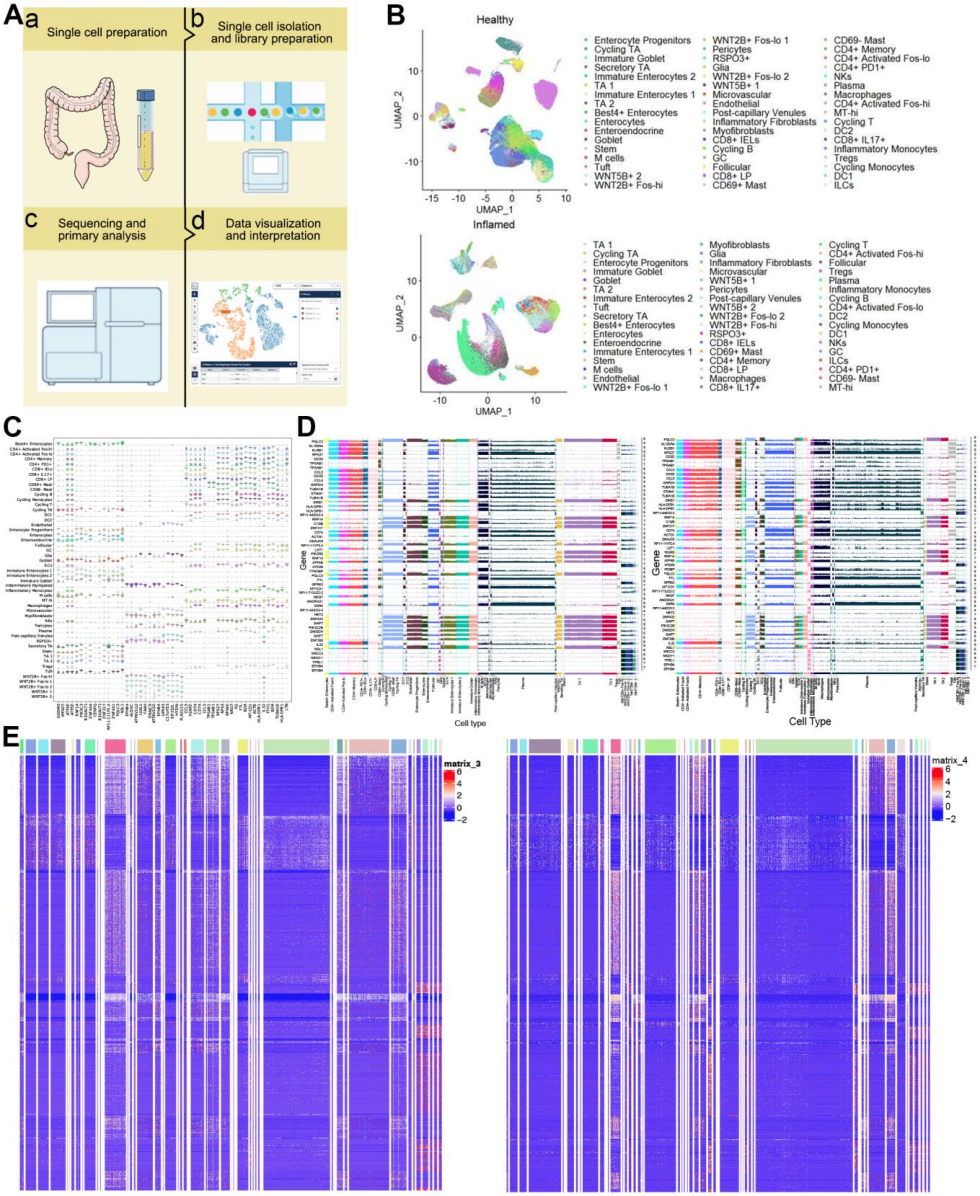
**Single-cell transcriptomic profiles from colon biopsies from UC patients and healthy individuals.** (**A**) Study design. (**B**) 2d visualization of 51 clusters of cells in healthy controls (up) and UC patients (down) on the UMAP plot. (**C**) Violin plots of specific marker genes in all types in healthy individuals. (**D**) Ridge plot. Expression of marker genes common to healthy individuals (left) and UC patients (right). (**E**) The heat map showing expression of UC-specific marker genes in healthy individuals (left) and UC patients (right). UMAP: uniform manifold approximation and projection for dimension reduction. UC: ulcerative colitis.

The sampling, sequencing and analysis workflow was show in Figure 1A. Through single-cell RNA sequencing of 68 colonoscopy specimens from 18 patients with UC and 12 healthy individuals, 366,650 high-quality cells were obtained. These cells were divided into 51 clusters based on the UMAP method. And we found that the distribution and number of cells of different subtypes was of difference (Figure 1B). The violin pictures of healthy individuals-specific marker genes for each cell type further supported these cell types (Figure 1C). In addition, the expression of marker genes common to UC patients and healthy controls was different in each cell type (Figure 1D). Furthermore, we identified marker genes specific to UC patients was whose expression differed in UC patients and healthy controls (Figure 1E and Supplementary Table 1).

### Immune cell landscape in UC

**Figure 2:**
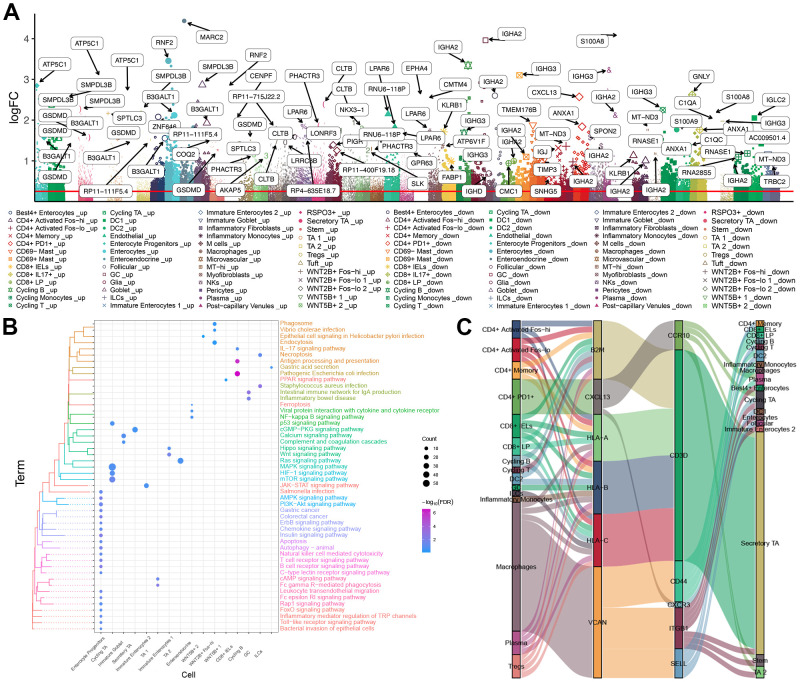
**Immune cell landscape in UC.** (**A**) Manhattan plot. The DEGs in 51 cell types between UC patients and healthy individuals. (**B**) Enrichment of DEGs in functional pathways of the Kyoto Encyclopedia of Genes and Genomes. (**C**) Sankey plot, showing communication among immune cells of different types via ligand-receptor pairs. DEGs: Differentially expressed genes. UC: ulcerative colitis.

UC impairs the integrity of the intestinal mucosa, which can compromise host immune tolerance toward intestinal microbes. Therefore we examined the landscape of immune cells in UC. Analysis of the DEGs in 51 types of cells (Figure 2A) showed functional enrichment in pathways involving immunity, apoptosis and inflammation, including pathways mediated by NF-κB, IL-17, PPAR, ErbB, and T cell receptors (Figure 2B). We also found that immune cells may communicate with each other via binding between surface receptors and their cognate ligands (Figure 2C). In particular, iTALK predicted that macrophages may communicate with Best4^+^ enterocytes, enterocytes and immature enterocytes (Supplementary Table 2). The interactions among the function pathways and genes in enterocytes of the above three subtypes were identified (Supplementary Tables 4–6). Interestingly, we found that some receptor or ligand genes (ITGB1, CD44, VCAN, CD4, ITGB2, AXL CANX and PLAUR) were significantly highly expressed in UC patients compared with healthy controls based on the bulk data (Supplementary Table 3).

### Single-cell atlas of ADMSCs

**Figure 3:**
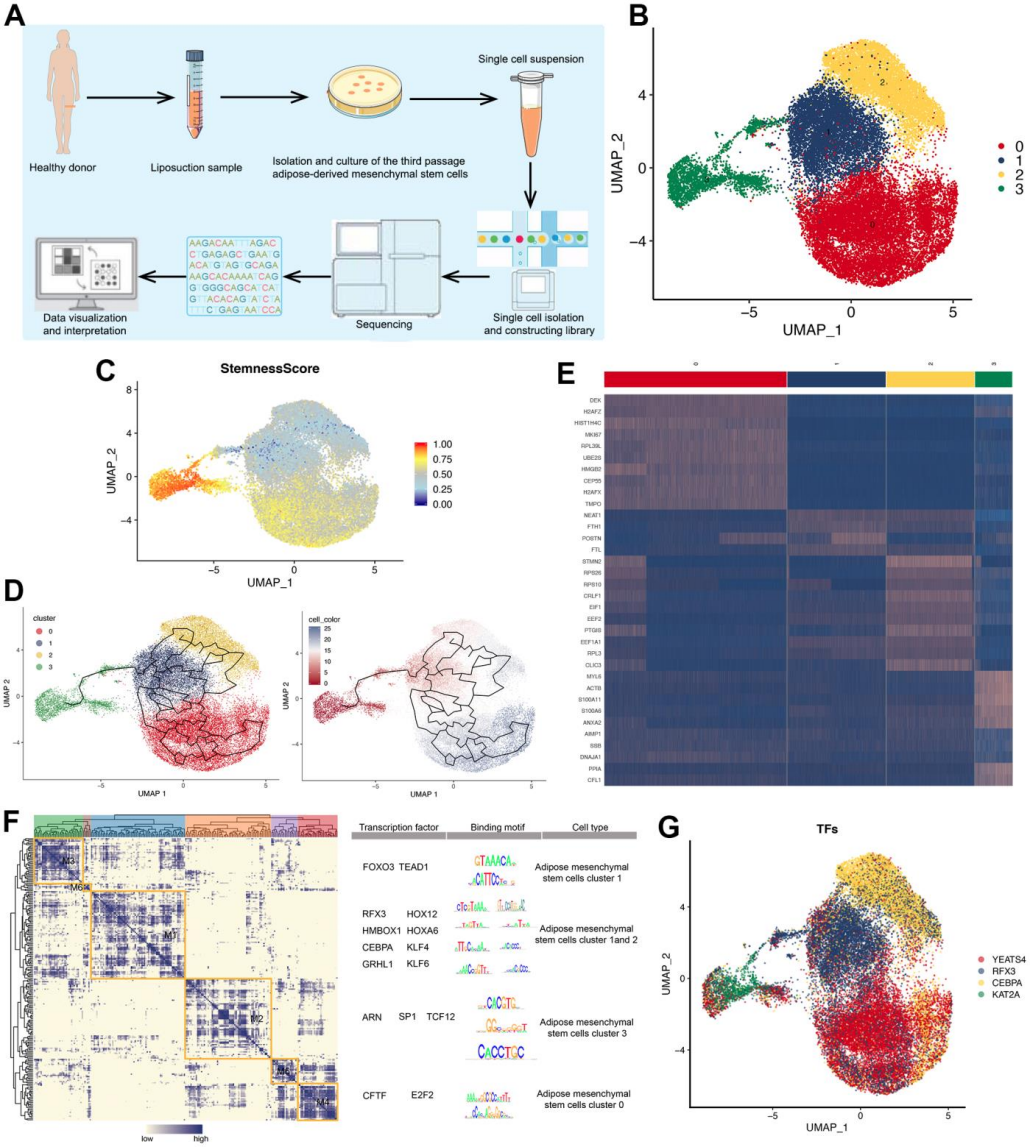
**Single-cell atlas of ADMSCs.** (**A**) Flow chart of sampling, sequencing and analysis of ADMSCs in this study. (**B**) 2D visualization of four clusters of 24,258 ADMSCs on the UMAP plot. (**C**) 2D visualization of stemness score of four cluster of ADMSCs on the UMAP plot. (**D**) Pseudotime developmental trajectory of ADMSCs shown in the UMAP plot. (**E**) Heat map, showing the expression of specific marker genes in each cluster of ADMSCs. (**F**) Regulon modules identified based on the regulon CSI matrix. The right panel shows representative transcription factors, their binding motifs, and associated cell types. (**G**) UMAP plot, showing the expression of transcription factors in each ADMSC cluster. ADMSCs: adipose-derived mesenchymal stem cells. UMAP: uniform manifold approximation and projection for dimension reduction. CSI: connection specificity index.

High-quality transcriptome data were obtained from 24,358 single ADMSCs (Figure 3A). UMAP dimensionality reduction showed that the cells were divided into four clusters (Figure 3B). The stemness score of 4 clusters was calculated, the Cluster 3 showed the highest stemness score, while clusters 1 and 2 showed the lowest (Figure 3C). Pseudo-time analysis suggested that ADMSCs in clusters 0, 1 and 2 differentiated from the cells in clusters 3 (Figure 3D).

The specific marker genes showed different expression patterns across the clusters (Figure 3E). Using the SCENIC method, we organized regulons of TFs with their most likely target genes into six modules. For each module, we identified several representative regulators and cell types based on average activity scores (Figure 3F), and UMAP analysis identified specific regulators in each cluster of ADMSCs (Figure 3G).

### Potential therapeutic mechanism of ADMSCs in UC

**Figure 4:**
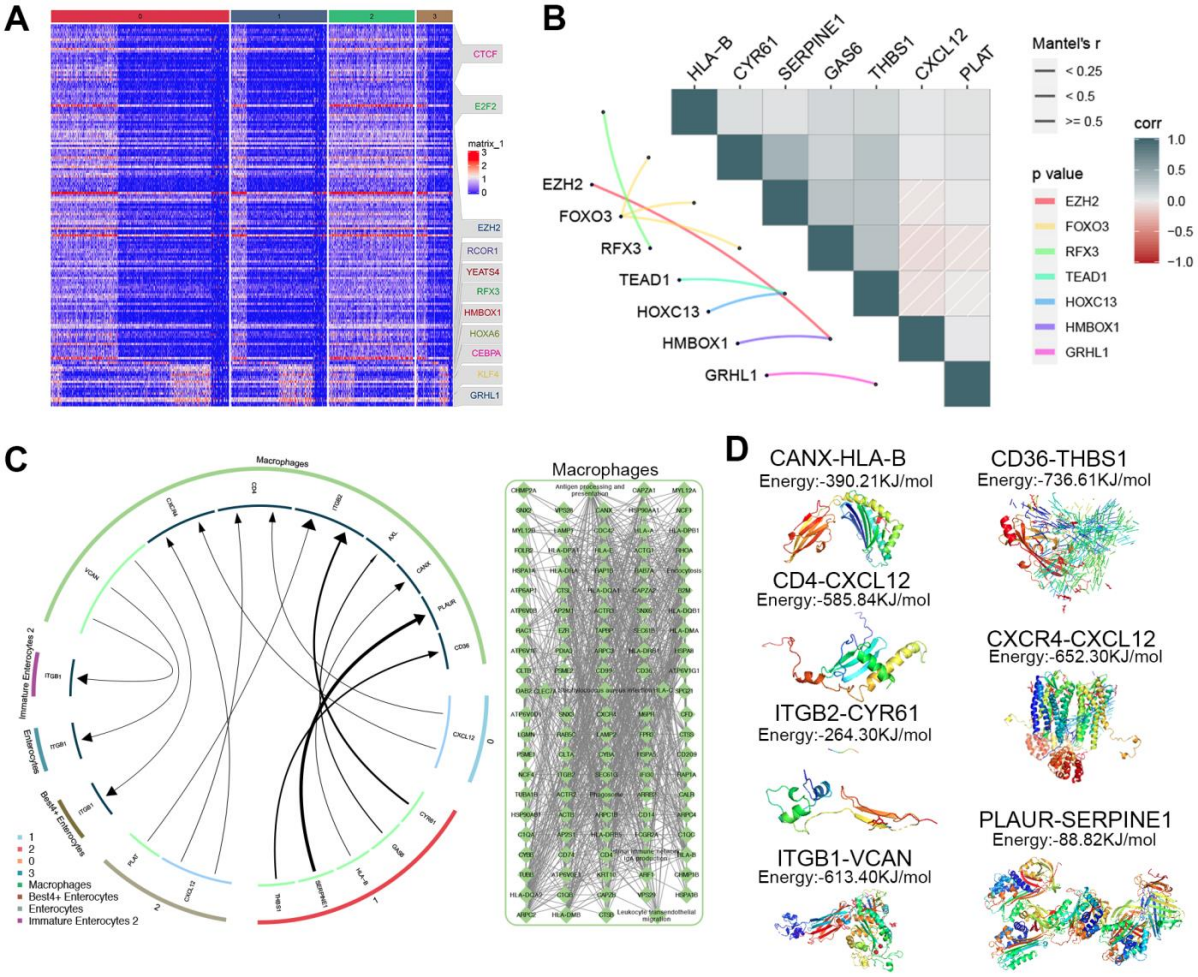
**Exploration of how ADMSCs may alleviate UC.** (**A**) Heat map, showing the expression of specific marker genes regulated by transcription factors in each ADMSCs cluster. (**B**) Correlation plot, showing relationships between transcription factors and ligands in ADMSCs. (**C**) Circos plot, showing relationships among macrophages, ADMSCs and three types of enterocytes. (**D**) Potential complexes between surface receptor and ligand obtained through molecular docking. ADMSCs: adipose-derived mesenchymal stem cells. UC:

**Figure 5:**
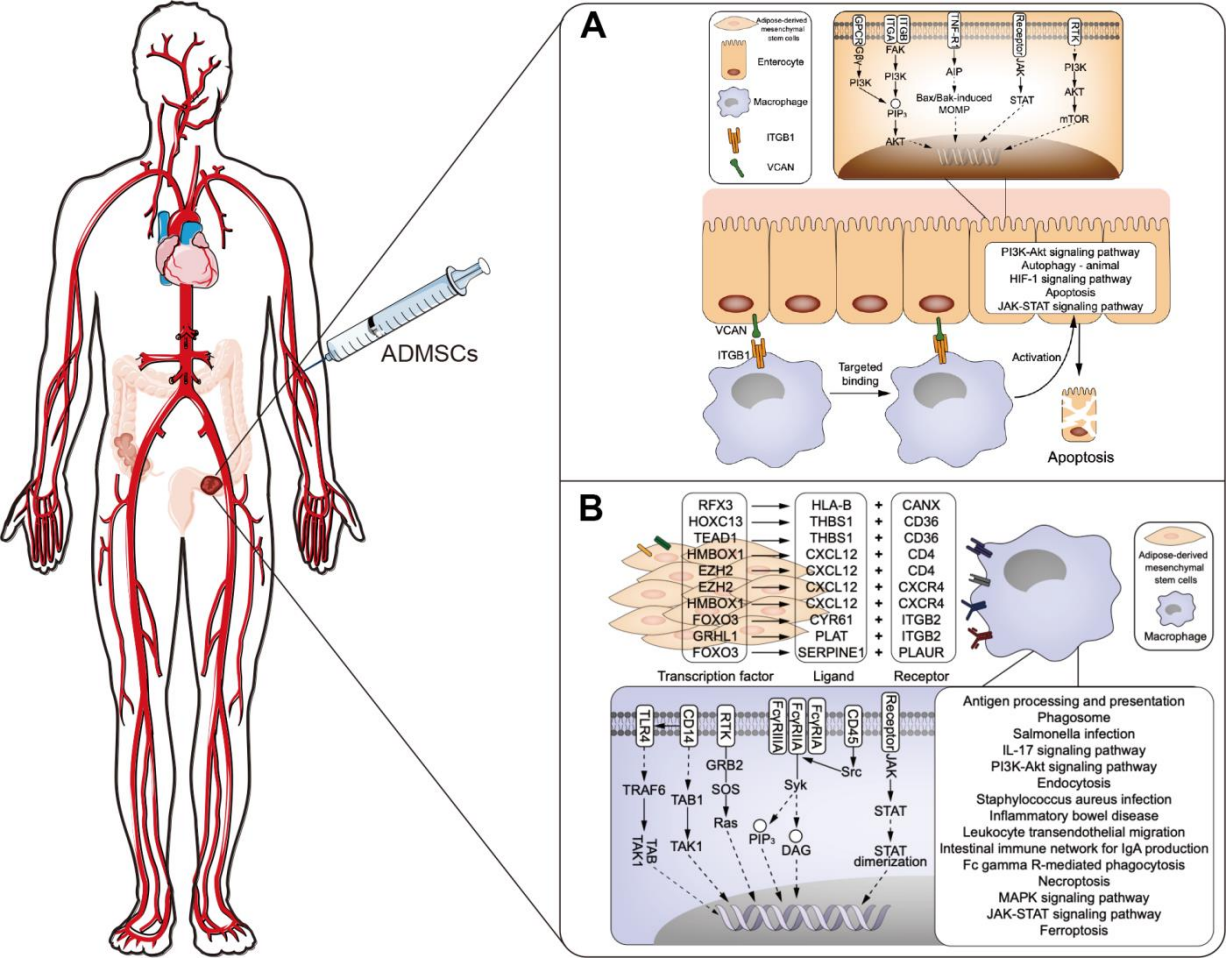
**Proposed mechanism about ADMSCs may alleviate UC.** (**A**) The proposed mechanism that macrophages may contribute to UC by communicating with enterocytes via ligand-receptor interactions. (**B**) Proposed mechanism of how ADMSCs may alleviate UC by communicating with macrophages and blocking inflammation. ADMSCs: adipose-derived mesenchymal stem cells. UC: ulcerative colitis.

Previous studies have suggested that ADMSCs can alleviate UC [16], but the mechanism involved remains unclear. We found that the expression pattern of genes targeted by regulons in each cluster of ADMSCs (Figure 4A). In addition, correlation analysis showed that expression level of regulons expression correlated with that of genes encoding surface ligand (Figure 4B). The iTALK analysis result suggested that ADMSCs may communicate with macrophages via ligand-receptor interactions. The result provided the evidence that macrophages may also communicate with Best 4^+^ enterocytes, enterocytes and immature enterocytes (Figure 4C). To examine the possibility of these cell-cell interactions via ligand-receptor interactions, molecular docking studies were performed, which indicated nine potential ligand-receptor pairs (Figure 4D). Based on the findings in this study, we proposed a mechanism that ADMSCs may alleviate UC after enter into the human body through communicating with macrophages, thereby further communicating with enterocytes by ligand-receptor pairs (Figure 5).

## DISCUSSION

In this bioinformatics study, we identified DEGs in different cell types of UC patients, which may participate in pathways involving immunity, apoptosis and inflammation. And we found that macrophages may communicate with enterocytes or ADMSCs. These results may help guide future research into the onset, progression and treatment of UC.

Enrichment results of the DEGs identified in our study of UC showed that the signaling pathways were significantly with immunity, apoptosis and inflammation. In this study, MAPK, Ras and mTOR signaling pathway, as well as *E. coli*-related biological function were significantly enriched based on the DEGs in UC patients. MAPK cascade in turn participates in cell migration, differentiation and proliferation [28]. In addition, Ras proteins function as molecular switches in signaling pathways involved in cell differentiation, growth, migration, survival and proliferation [29].

mTOR, a highly conserved serine/threonine protein kinase that regulates several biological processes [30]. Enteropathogenic *E.coli* (EPEC) and enterohemorrhagic *E.coli* (EHEC) are closely related pathogenic strains of *Escherichia coli* [31], which damage enterocytes and may increase risk of UC [32].

UC involves extensive damage to enterocytes and diffuse inflammation [2]. Macrophages interact with enterocytes [33] and may compromise gap junctions between enterocytes during intestinal inflammation [34]. Our iTALK analysis suggests cross-talk between them, implying that the interaction between macrophages and enterocytes may contribute to UC. Our results further suggest that ADMSCs communicate with macrophages to block inflammation and thereby alleviate UC. These *in silico* findings are consistent with biological studies showing that ADMSCs interact with immune cells [35] and can shift macrophages from a pro-inflammatory M1 phenotype to an anti-inflammatory M2 phenotype [36].

Using the SCENIC method, we proposed a gene regulatory network in ADMSCs. Module M1 in the network contains regulators of gene expression, morphogenesis, and differentiation, and it also contains genes encoding zinc finger proteins required for normal development of the epithelial barrier, such as HOX12 [37], HOXA6 [38], KLF4 [39] and KLF6 [40]. Module M2 contains the regulators SP1, which is involved in many cellular processes [41], and TCF12, which is involved in cell cycle regulation or DNA replication [42]. Module M3 contains regulators FOXO3 and TEAD1. FOXO3 likely activates genes that promote apoptosis and autophagy [43, 44], while the TF TEAD1 may promote apoptosis and restrict proliferation [45]. The consistency between our bioinformatics analyses of ADMSCs and previous biological studies suggests the reliability of our approach.

There are some limitations in this study. On the other hand, the data in this study come from a relatively small sample and were analyzed using only bioinformatics techniques, but they provide a useful reference for experimental work to clarify the pathogenesis of UC and develop effective treatments. On the other hand, the expression of genes in UC patients and healthy individuals was preliminarily validated on the bulk data level, the findings in this study needs to be validated in more bulk data or the biological experiments.

## Supplementary Material

Supplementary Table 1

Supplementary Tables 2 and 3

Supplementary Table 4

Supplementary Table 5

Supplementary Table 6

## References

[1] Smillie CS, Biton M, Ordovas-Montanes J, Sullivan KM, Burgin G, Graham DB, Herbst RH, Rogel N, Slyper M, Waldman J, Sud M, Andrews E, Velonias G, et al. Intra- and Inter-cellular Rewiring of the Human Colon during Ulcerative Colitis. Cell. 2019; 178:714–30.e22. 10.1016/j.cell.2019.06.029 31348891 PMC6662628

[2] Ordás I, Eckmann L, Talamini M, Baumgart DC, Sandborn WJ. Ulcerative colitis. Lancet. 2012; 380:1606–19. 10.1016/S0140-6736(12)60150-0 22914296

[3] Yang C, Merlin D. Nanoparticle-Mediated Drug Delivery Systems For The Treatment Of IBD: Current Perspectives. Int J Nanomedicine. 2019; 14:8875–89. 10.2147/IJN.S210315 32009785 PMC6859086

[4] Burge K, Gunasekaran A, Eckert J, Chaaban H. Curcumin and Intestinal Inflammatory Diseases: Molecular Mechanisms of Protection. Int J Mol Sci. 2019; 20:1912. 10.3390/ijms20081912 31003422 PMC6514688

[5] Salvo Romero E, Alonso Cotoner C, Pardo Camacho C, Casado Bedmar M, Vicario M. The intestinal barrier function and its involvement in digestive disease. Rev Esp Enferm Dig. 2015; 107:686–96. 10.17235/reed.2015.3846/2015 26541659

[6] Wang J, Zhang C, Guo C, Li X. Chitosan Ameliorates DSS-Induced Ulcerative Colitis Mice by Enhancing Intestinal Barrier Function and Improving Microflora. Int J Mol Sci. 2019; 20:5751. 10.3390/ijms20225751 31731793 PMC6888260

[7] Groschwitz KR, Hogan SP. Intestinal barrier function: molecular regulation and disease pathogenesis. J Allergy Clin Immunol. 2009; 124:3–20. 10.1016/j.jaci.2009.05.038 19560575 PMC4266989

[8] Ge J, Li YJ, Liu AQ, Huang WY, Yang F, Ma L, Zhai HH. [Changes of intestinal mucosal barrier in mice with chronic ulcerative colitis]. Zhonghua Yi Xue Za Zhi. 2018; 98:3950–3. 10.3760/cma.j.issn.0376-2491.2018.48.009 30669801

[9] Tatiya-Aphiradee N, Chatuphonprasert W, Jarukamjorn K. Immune response and inflammatory pathway of ulcerative colitis. J Basic Clin Physiol Pharmacol. 2018; 30:1–10. 10.1515/jbcpp-2018-0036 30063466

[10] Porter RJ, Kalla R, Ho GT. Ulcerative colitis: Recent advances in the understanding of disease pathogenesis. F1000Res. 2020; 9:F1000. 10.12688/f1000research.20805.1 32399194 PMC7194476

[11] Gren ST, Grip O. Role of Monocytes and Intestinal Macrophages in Crohn’s Disease and Ulcerative Colitis. Inflamm Bowel Dis. 2016; 22:1992–8. 10.1097/MIB.0000000000000824 27243595

[12] Zhao H, Shang Q, Pan Z, Bai Y, Li Z, Zhang H, Zhang Q, Guo C, Zhang L, Wang Q. Exosomes From Adipose-Derived Stem Cells Attenuate Adipose Inflammation and Obesity Through Polarizing M2 Macrophages and Beiging in White Adipose Tissue. Diabetes. 2018; 67:235–47. 10.2337/db17-0356 29133512

[13] Liu X, Xiang Q, Xu F, Huang J, Yu N, Zhang Q, Long X, Zhou Z. Single-cell RNA-seq of cultured human adipose-derived mesenchymal stem cells. Sci Data. 2019; 6:190031. 10.1038/sdata.2019.31 30806636 PMC6390702

[14] Cheng J, Zhang J, Wu Z, Sun X. Inferring microenvironmental regulation of gene expression from single-cell RNA sequencing data using scMLnet with an application to COVID-19. Brief Bioinform. 2021; 22:988–1005. 10.1093/bib/bbaa327 33341869 PMC7799217

[15] Liu Z, Sun Z, Liu H, Niu W, Wang X, Liang N, Wang X, Wang Y, Shi Y, Xu L, Shi W. Single-cell transcriptomic analysis of eutopic endometrium and ectopic lesions of adenomyosis. Cell Biosci. 2021; 11:51. 10.1186/s13578-021-00562-z 33685511 PMC7938473

[16] Yu G, Wang LG, Han Y, He QY. clusterProfiler: an R package for comparing biological themes among gene clusters. OMICS. 2012; 16:284–7. 10.1089/omi.2011.0118 22455463 PMC3339379

[17] Liberzon A, Subramanian A, Pinchback R, Thorvaldsdóttir H, Tamayo P, Mesirov JP. Molecular signatures database (MSigDB) 3.0. Bioinformatics. 2011; 27:1739–40. 10.1093/bioinformatics/btr260 21546393 PMC3106198

[18] Hänzelmann S, Castelo R, Guinney J. GSVA: gene set variation analysis for microarray and RNA-seq data. BMC Bioinformatics. 2013; 14:7. 10.1186/1471-2105-14-7 23323831 PMC3618321

[19] Sun T, Liu Z, Yang Q. The role of ubiquitination and deubiquitination in cancer metabolism. Mol Cancer. 2020; 19:146. 10.1186/s12943-020-01262-x 33004065 PMC7529510

[20] Aibar S, González-Blas CB, Moerman T, Huynh-Thu VA, Imrichova H, Hulselmans G, Rambow F, Marine JC, Geurts P, Aerts J, van den Oord J, Atak ZK, Wouters J, Aerts S. SCENIC: single-cell regulatory network inference and clustering. Nat Methods. 2017; 14:1083–6. 10.1038/nmeth.4463 28991892 PMC5937676

[21] Davie K, Janssens J, Koldere D, De Waegeneer M, Pech U, Kreft Ł, Aibar S, Makhzami S, Christiaens V, Bravo González-Blas C, Poovathingal S, Hulselmans G, Spanier KI, et al. A Single-Cell Transcriptome Atlas of the Aging Drosophila Brain. Cell. 2018; 174:982–98.e20. 10.1016/j.cell.2018.05.057 29909982 PMC6086935

[22] Han X, Wang R, Zhou Y, Fei L, Sun H, Lai S, Saadatpour A, Zhou Z, Chen H, Ye F, Huang D, Xu Y, Huang W, et al. Mapping the Mouse Cell Atlas by Microwell-Seq. Cell. 2018; 172:1091–107.e17. 10.1016/j.cell.2018.02.001 29474909

[23] Planell N, Lozano JJ, Mora-Buch R, Masamunt MC, Jimeno M, Ordás I, Esteller M, Ricart E, Piqué JM, Panés J, Salas A. Transcriptional analysis of the intestinal mucosa of patients with ulcerative colitis in remission reveals lasting epithelial cell alterations. Gut. 2013; 62:967–76. 10.1136/gutjnl-2012-303333 23135761

[24] Ritchie ME, Phipson B, Wu D, Hu Y, Law CW, Shi W, Smyth GK. limma powers differential expression analyses for RNA-sequencing and microarray studies. Nucleic Acids Res. 2015; 43:e47. 10.1093/nar/gkv007 25605792 PMC4402510

[25] Macindoe G, Mavridis L, Venkatraman V, Devignes MD, Ritchie DW. HexServer: an FFT-based protein docking server powered by graphics processors. Nucleic Acids Res. 2010; 38:W445–9. 10.1093/nar/gkq311 20444869 PMC2896144

[26] Burley SK, Berman HM, Kleywegt GJ, Markley JL, Nakamura H, Velankar S. Protein Data Bank (PDB): The Single Global Macromolecular Structure Archive. Methods Mol Biol. 2017; 1607:627–41. 10.1007/978-1-4939-7000-1_26 28573592 PMC5823500

[27] Mooers BH. Shortcuts for faster image creation in PyMOL. Protein Sci. 2020; 29:268–76. 10.1002/pro.3781 31710740 PMC6933860

[28] Chen Z, Gibson TB, Robinson F, Silvestro L, Pearson G, Xu B, Wright A, Vanderbilt C, Cobb MH. MAP kinases. Chem Rev. 2001; 101:2449–76. 10.1021/cr000241p 11749383

[29] Karnoub AE, Weinberg RA. Ras oncogenes: split personalities. Nat Rev Mol Cell Biol. 2008; 9:517–31. 10.1038/nrm2438 18568040 PMC3915522

[30] Kennedy BK, Lamming DW. The Mechanistic Target of Rapamycin: The Grand ConducTOR of Metabolism and Aging. Cell Metab. 2016; 23:990–1003. 10.1016/j.cmet.2016.05.009 27304501 PMC4910876

[31] Pinaud L, Sansonetti PJ, Phalipon A. Host Cell Targeting by Enteropathogenic Bacteria T3SS Effectors. Trends Microbiol. 2018; 26:266–83. 10.1016/j.tim.2018.01.010 29477730

[32] Santos AS, Finlay BB. Bringing down the host: enteropathogenic and enterohaemorrhagic Escherichia coli effector-mediated subversion of host innate immune pathways. Cell Microbiol. 2015; 17:318–32. 10.1111/cmi.12412 25588886

[33] Liu H, Kai L, Du H, Wang X, Wang Y. LPS Inhibits Fatty Acid Absorption in Enterocytes through TNF-α Secreted by Macrophages. Cells. 2019; 8:1626. 10.3390/cells8121626 31842409 PMC6953048

[34] Anand RJ, Dai S, Rippel C, Leaphart C, Qureshi F, Gribar SC, Kohler JW, Li J, Stolz DB, Sodhi C, Hackam DJ. Activated macrophages inhibit enterocyte gap junctions via the release of nitric oxide. Am J Physiol Gastrointest Liver Physiol. 2008; 294:G109–19. 10.1152/ajpgi.00331.2007 17975131

[35] Fang B, Song Y, Liao L, Zhang Y, Zhao RC. Favorable response to human adipose tissue-derived mesenchymal stem cells in steroid-refractory acute graft-versus-host disease. Transplant Proc. 2007; 39:3358–62. 10.1016/j.transproceed.2007.08.103 18089385

[36] Sun M, Sun L, Huang C, Chen BC, Zhou Z. Induction of Macrophage M2b/c Polarization by Adipose Tissue-Derived Mesenchymal Stem Cells. J Immunol Res. 2019; 2019:7059680. 10.1155/2019/7059680 31321244 PMC6607735

[37] Walters JR, Howard A, Rumble HE, Prathalingam SR, Shaw-Smith CJ, Legon S. Differences in expression of homeobox transcription factors in proximal and distal human small intestine. Gastroenterology. 1997; 113:472–7. 10.1053/gast.1997.v113.pm9247466 9247466

[38] Wu F, Wu S, Tong H, He W, Gou X. HOXA6 inhibits cell proliferation and induces apoptosis by suppressing the PI3K/Akt signaling pathway in clear cell renal cell carcinoma. Int J Oncol. 2019; 54:2095–105. 10.3892/ijo.2019.4789 31081053 PMC6521939

[39] Sousa L, Pankonien I, Simões FB, Chanson M, Amaral MD. Impact of KLF4 on Cell Proliferation and Epithelial Differentiation in the Context of Cystic Fibrosis. Int J Mol Sci. 2020; 21:6717. 10.3390/ijms21186717 32937756 PMC7555189

[40] Syafruddin SE, Rodrigues P, Vojtasova E, Patel SA, Zaini MN, Burge J, Warren AY, Stewart GD, Eisen T, Bihary D, Samarajiwa SA, Vanharanta S. A KLF6-driven transcriptional network links lipid homeostasis and tumour growth in renal carcinoma. Nat Commun. 2019; 10:1152. 10.1038/s41467-019-09116-x 30858363 PMC6411998

[41] Rowbotham K, Haugen J, Milavetz B. Differential SP1 interactions in SV40 chromatin from virions and minichromosomes. Virology. 2020; 548:124–31. 10.1016/j.virol.2020.06.012 32838933 PMC10035769

[42] Zhang R, Wang L, Pan JH, Han J. A critical role of E2F transcription factor 2 in proinflammatory cytokines-dependent proliferation and invasiveness of fibroblast-like synoviocytes in rheumatoid Arthritis. Sci Rep. 2018; 8:2623. 10.1038/s41598-018-20782-7 29422529 PMC5805761

[43] Brunet A, Bonni A, Zigmond MJ, Lin MZ, Juo P, Hu LS, Anderson MJ, Arden KC, Blenis J, Greenberg ME. Akt promotes cell survival by phosphorylating and inhibiting a Forkhead transcription factor. Cell. 1999; 96:857–68. 10.1016/s0092-8674(00)80595-4 10102273

[44] Lehtinen MK, Yuan Z, Boag PR, Yang Y, Villén J, Becker EB, DiBacco S, de la Iglesia N, Gygi S, Blackwell TK, Bonni A. A conserved MST-FOXO signaling pathway mediates oxidative-stress responses and extends life span. Cell. 2006; 125:987–1001. 10.1016/j.cell.2006.03.046 16751106

[45] Tome-Garcia J, Erfani P, Nudelman G, Tsankov AM, Katsyv I, Tejero R, Zhang B, Walsh M, Friedel RH, Zaslavsky E, Tsankova NM. Analysis of chromatin accessibility uncovers TEAD1 as a regulator of migration in human glioblastoma. Nat Commun. 2018; 9:4020. 10.1038/s41467-018-06258-2 30275445 PMC6167382

