# Supplementary material for: Adipose-derived mesenchymal stem cells may reduce intestinal epithelial damage in ulcerative colitis by communicating with macrophages and blocking inflammatory pathways: an analysis in silico
Source: Aging (Albany NY). 2022 Mar 22;14(6):2665–77. doi: 10.18632/aging.203964 (PMC9004563; doi:10.18632/aging.203964)
Supplement: Supplementary Tables 2 and 3 [file aging-14-203964-s002.pdf]

SUPPLEMENTARY TABLES

Supplementary Table 2. The receptor and ligand pairs of macrophages and enterocytes of different subtypes.

Ligand	Receptor	Cell_from_mean_exprs	Cell_from	Cell_to_mean_exprs	Cell_to
VIM	CD44	392.9345568	1	0.639843532	Macrophages
COL1A1	CD44	214.0378432	1	0.639843532	Macrophages
COL1A2	CD44	117.4523317	2	0.639843532	Macrophages
FN1	CD44	76.85261818	2	0.639843532	Macrophages
COL1A1	CD36	135.694858	0	0.340597932	Macrophages
APP	CD74	1.95886416	0	17.00223526	Macrophages
COL1A1	CD44	31.21820918	3	0.639843532	Macrophages
FN1	PLAUR	26.22723853	3	0.566359318	Macrophages
PKM	CD44	23.19493477	0	0.639843532	Macrophages
VCAN	CD44	7.218096458	1	0.639843532	Macrophages
FGF2	CD44	4.701765157	0	0.639843532	Macrophages
THBS1	CD36	6.203453569	0	0.340597932	Macrophages
CTGF	ITGB2	2.679834462	3	0.664291702	Macrophages
SPON2	ITGB2	2.673404425	2	0.664291702	Macrophages
ANXA1	FPR3	6.580932675	2	0.250209556	Macrophages
TGFB1	CXCR4	4.411153791	1	0.293657446	Macrophages
THBS1	CD36	3.579759217	3	0.340597932	Macrophages
GAS6	AXL	7.75743349	1	0.134534786	Macrophages
GAS6	AXL	4.842440522	0	0.134534786	Macrophages
HLA-B	CANX	1.37237145	0	0.35582565	Macrophages
CXCL12	CXCR4	1.493169609	0	0.293657446	Macrophages
CSF1	CSF1R	0.836543053	2	0.289466331	Macrophages
CSF1	CSF1R	0.587797391	0	0.289466331	Macrophages
PROS1	AXL	0.358109184	2	0.134534786	Macrophages
ICAM3	ITGB2	0.062829195	3	0.664291702	Macrophages
VCAN	ITGB1	0.654512434	Macrophages	0.462867012	Best4+ Enterocytes
VCAN	ITGB1	0.654512434	Macrophages	0.4375	Enterocytes
VCAN	ITGB1	0.654512434	Macrophages	0.498440424	Immature Enterocytes 2

Supplementary Table 3. The expression levels of the differentially expressed receptor or ligand genes obtained in bulk data through single cell analysis.

Symbol	LogFC	AveExpr	t	P.Value	adj.P.Val	B
ITGB1	0.014940634	3.782862	2.2946137	0.026785722	0.146494886	-4.382364
CD44	0.1351186	3.507543	5.180855	5.81345E-06	0.000540562	3.604434
VCAN	0.2244977	2.672022	3.40536	0.001458855	0.01869735	-1.709587
CD4	0.08734719	2.941532	2.664814	1.09E-02	0.077302676	-3.5718828
ITGB2	0.22633679	3.022593	3.041501	0.004032696	0.03837442	-2.66088
AXL	0.150793	2.925003	2.495249	0.01657418	0.1042333	-3.954259
CANX	0.04339978	3.655007	5.314411	3.75E-06	0.000410103	4.031727
PLAUR	0.1633127	2.991897	3.309774	0.001915449	0.02262415	-1.96566
CD36	-0.1378495	2.660956	-2.070367	0.04456048	0.2074994	-4.827498
HLA-A	-0.003592468	3.834781	-0.467279	0.6426995	0.9354278	-6.784791
HLA-B	-0.01278865	3.844941	-0.8690066	0.3897497	0.7887466	-6.515917
HLA-C	-0.01500723	3.70149	-0.9425641	0.3512585	0.7486185	-6.449699
CXCR4	0.1313991	3.17712	1.456362	0.1526787	0.4684196	-5.846819
